# Estrogenic activity, race/ethnicity, and Indigenous American ancestry among San Francisco Bay Area women

**DOI:** 10.1371/journal.pone.0213809

**Published:** 2019-03-25

**Authors:** Sylvia S. Sanchez, Phum Tachachartvanich, Frank Z. Stanczyk, Scarlett L. Gomez, Esther M. John, Martyn T. Smith, Laura Fejerman

**Affiliations:** 1 Division of Environmental Health Sciences, School of Public Health, University of California, Berkeley, Berkeley, California, United States of America; 2 Department of Obstetrics and Gynecology, Keck School of Medicine, University of Southern California, Los Angeles, California, United States of America; 3 Department of Medicine, Division of Oncology and Stanford Cancer Institute, Stanford School of Medicine, Stanford, California, United States of America; 4 Department of Epidemiology and Biostatistics, Helen Diller Comprehensive Cancer Center, University of California, San Francisco, San Francisco, California, United States of America; 5 Division of General Internal Medicine, Institute of Human Genetics, Helen Diller Comprehensive Cancer Center and Department of Medicine, University of California, San Francisco, San Francisco, California, United States of America; Universita degli Studi di Padova, ITALY

## Abstract

Estrogens play a significant role in breast cancer development and are not only produced endogenously, but are also mimicked by estrogen-like compounds from environmental exposures. We evaluated associations between estrogenic (E) activity, demographic factors and breast cancer risk factors in Non-Latina Black (NLB), Non-Latina White (NLW), and Latina women. We examined the association between E activity and Indigenous American (IA) ancestry in Latina women. Total E activity was measured with a bioassay in plasma samples of 503 women who served as controls in the San Francisco Bay Area Breast Cancer Study. In the univariate model that included all women with race/ethnicity as the independent predictor, Latinas had 13% lower E activity (p = 0.239) and NLBs had 35% higher activity (p = 0.04) compared to NLWs. In the multivariable model that adjusted for demographic factors, Latinas continued to show lower E activity levels (26%, p = 0.026), but the difference between NLBs and NLWs was no longer statistically significant (p = 0.431). An inverse association was observed between E activity and IA ancestry among Latina women (50% lower in 0% vs. 100% European ancestry, p = 0.027) consistent with our previously reported association between IA ancestry and breast cancer risk. These findings suggest that endogenous estrogens and exogenous estrogen-like compounds that act on the estrogen receptor and modulate E activity may partially explain racial/ethnic differences in breast cancer risk.

## Introduction

Epidemiological studies have consistently reported that endogenous sex hormones play a critical role in the etiology of numerous diseases including breast cancer [1–4]. Interestingly, among U.S. racial/ethnic groups the reported variation in endogenous hormone levels is consistent with observed racial/ethnic differences in the incidence of breast cancer [5–7]. For example, in the Women’s Health Initiative Dietary Modification Trial, African American women had significantly higher concentrations of endogenous reproductive hormones compared to non-Latina White women [8], whereas higher levels of urinary concentrations of estrogens were strongly associated with breast cancer risk in Asian women in the Shanghai Women’s Study cohort [9]. Moreover, in the Multiethnic Cohort study, postmenopausal Native Hawaiian and African American women tended to have higher levels of endogenous hormones when compared to non-Latina Whites (NLWs) [10], while foreign-born Hispanic/Latina women (referred to as Latinas hereafter) had lower hormone levels which correlates with their lower incidence of breast cancer [5].

Changes in lifestyle and demographic factors can influence endogenous estrogen levels through changes in adiposity. Across racial/ethnic groups, multiple studies have consistently identified a positive association between body mass index (BMI) and estrogen levels [11], [12]. In Latina women, adaptation of a Western lifestyle, along with greater physical inactivity have served as potential explanations for changes in BMI [13]. Estrogen plays a significant role in breast cancer and is not only produced endogenously, but is also mimicked by exogenous sources including xenoestrogens and phytoestrogens [14–16] like bisphenol A (BPA), diethylstilbestrol (DES), atrazine, and soy products. It is because of this dual role that endogenous and exogenous sources could contribute to differences in breast cancer incidence rates in different racial/ethnic populations across the U.S. [17].

Various methods are available to quantify estrogen receptor (ER) function and activation. Luciferase assays have been widely used and have proven useful in predicting breast cancer risk [18], [19]. Generally, due to the fluctuations of estrogens throughout the menstrual cycle, estrogen measures have been more consistent for postmenopausal than premenopausal women [20].

In a previous pilot study among foreign-born and U.S.-born Mexican women who participated as controls in a population-based case-control study of breast cancer, we found that plasma estrogenic (E) activity was associated with genetic ancestry and years of U.S. residence [18], suggesting the possibility of a hormone related pathway for the observed racial/ethnic differences in breast cancer risk. In the present study, we examined total E activity accounting for both endogenous and exogenous sources of estrogenic compounds in plasma of 503 women, the majority of them postmenopausal, from three racial/ethnic groups. We hypothesize that differences in breast cancer risk in different racial/ethnic groups could be partly due to variations in endogenous estrogens and exogenous estrogen-like exposures. We evaluated the association between E activity, demographic and lifestyle factors in non-Latina Black (NLB), NLW, and Latina women, accounting for endogenous estrogen levels. We also used a larger sample of Latina women to validate the previously observed association between E activity and Indigenous American (IA) ancestry [18]. The approach we used provides an efficient way to assess endogenous and exogenous exposures and provides insights on a mechanistic connection that explains the differences in breast cancer incidence rates in different racial/ethnic populations living in the US.

## Materials and methods

### Study samples/ control selection

Participants were selected from the control group of the San Francisco Bay Area Breast Cancer Study (SFBCS), a population-based case-control study in Latina, NLB, and NLW women [13]. Controls living in San Francisco, San Mateo, Alameda, Contra Costa or Santa Clara counties were identified through random-digit dialing and frequency matched to breast cancer cases diagnosed from 1995–2002 in the same counties on race/ethnicity and 5-year age group. Blood samples were collected for a subset of study participants (cases diagnosed from 1997–2002 and their matched controls). Professional trained interviewers administered a questionnaire on breast cancer risk factors in English or Spanish at a home visit and took anthropometric measurements. Blood samples were collected shortly after the interview and in some instances a few years after the interview as part of an ancillary study. A short questionnaire was administered at time of blood draw to obtain updates on key variables (current menstrual status and weight; pregnancy, breast-feeding, smoking, alcohol consumption, and medication use in the previous 6 months).

In the present study, we measured E activity in plasma samples of 503 women (329 Latinas, 100 NLBs, and 74 NLWs) from the control group of SFBCS. Eight percent of the participants were premenopausal and equally distributed among the different racial/ethnic groups. To prevent exposure misclassification of exogenous estrogen use, we excluded women who were using hormone therapy at the time of blood draw. Participants who had one or more missing covariates were excluded from the analyses (11 women in analyses without adjustment for endogenous estrogens; 15 women in analyses with adjustment for endogenous estrogens).

All study participants provided written informed consent. The study was approved by the Institutional Review Boards at the University of California, San Francisco and the Cancer Prevention Institute of California.

### Endogenous hormone measurements

Plasma hormone measurements were performed at the Reproductive Endocrine Research laboratory at the University of Southern California under the direction of one of the contributing authors (FZS). Methods are described elsewhere [5]. Briefly, plasma concentrations of estrone and bioavailable estradiol were obtained by a radioimmunoassay method after organic solvent extraction and Celite column partition chromatography. Reported endogenous estrogen levels are the sum of estrone and bioavailable estradiol in free and albumin bound form. The assay sensitivities for estrone and estradiol are 4 pg/ml and 2 pg/ml, respectively, and the inter-assay coefficient of variation (CV) for each assay is less than 12%.

### Estrogenic activity measurement

We utilized a receptor-mediated Chemical-Activated Luciferase gene eXpression (CALUX) assay for the assessment of total E activity profiles in human plasma, which captures levels of both endogenous and exogenous estrogenic compounds. Methods followed procedures as previously described [18], [21]. Briefly, endogenous and exogenous estrogens and estrogen-like compounds (e.g., 17-beta estradiol (E2), phytoestrogens, 4-nonylphenol) in human plasma were measured using a transfected human breast cancer cell line with luciferase reporter, T47D-Kbluc (ATCC, Manassas, VA). Cells were cultured in phenol red DMEM with 10% FBS until one week prior to cell treatment with plasma. Cells were placed in phenol red free DMEM media with 10% charcoal dextran serum to ensure removal of all external sources of estrogens for one week prior to plasma addition. After one week in phenol red free medium, cells between passage 10 and 16 were seeded at a density of 27,000 cells per well and a final volume of 200 uL per well in 96-well microtiter plates and incubated at 37 degrees Celsius. After a 24 hour incubation period, 8uL of plasma per sample was diluted in phenol red free medium and added in quadruplicates directly onto the cells. A second incubation period of 24 hours at 37 degrees Celsius followed plasma addition. Lastly, cells were lysed using 1X passive lysis buffer (Promega, Madison, WI, USA) and results were obtained using a microplate luminometer (Berthold Technologies, Oak Ridge, TN, USA). Reporter activity was measured by the fluorescence emitted per well. Results were expressed in relative light units (RLUs) with higher RLU values reflecting greater E activity in plasma. Four RLU readings per sample were averaged to express results as one measure. The measurement was converted to picomolar (pM) equivalents based on the standard curve of (17β)-estra-1,3,5(10)-triene-3,17-diol, or 17β-estradiol (Tocris Bioscience, Bristol, UK) on each plate expanding a range of 0.0 pM to 25.0 pM. Samples from each racial/ethnic group were included within each batch. Lab personnel were blinded to the race/ethnicity of the samples. The minimum limit of detection for estradiol is 1.0 pM and the intra-assay (technical) and inter-assay (biological) CVs of this assay are between 7–23%, with higher values often corresponding to premenopausal women.

### Statistical methods

All hormone values were natural log (ln) transformed to approximate normal distribution. Differences in means or proportions for all analyzed variables between racial/ethnic groups were assessed using two-sided t-tests and Fisher’s exact tests, respectively. Linear regression was used to analyze the relationship between ln-transformed and plate adjusted E activity (dependent variable) and race/ethnicity (main predictor). Plate adjusted values were obtained by first estimating average plate effects using linear regression and then subtracting the average plate effect from each individual value. Percent change in RLUs per unit change of predictor variables was calculated using the formula [e^β^-1]*100. Statistical significance was set at p<0.05. All statistical analyses were performed using the program Stata [22].

The present study assessed differences in E activity by race/ethnicity and tested associations between E activity and various factors that were hypothesized to affect E activity levels (i.e., height, BMI, age, alcohol intake, and neighborhood SES). Multivariable regression models included several independent variables such as race/ethnicity (Latina, NLW, NLB), age at blood draw (categorical, <55, 55 to 65 and ≥ 66 years), height (continuous, in centimeters), body mass index (BMI) at blood draw (categorical, <25, 25–29.9, and ≥30 kg/m^2^), neighborhood socioeconomic status (SES) (categorical, 1 = low SES, 5 = high SES) geocoded to a Census 2000 block group, alcohol intake during the calendar year before selection into the parent study (categorical, grams per day), and E activity (continuous, log-transformed). Selection of these variables was based on factors we previously hypothesized could affect E activity at time of blood draw [18]. BMI was calculated as self-reported weight (kg) at blood draw / height squared (m) measured at interview. For a subset of Latina women (n = 276), information was available on IA ancestry. Proportion of IA ancestry was used as a continuous variable with values ranging from 0 to 100%. The multivariable model for the ancestry analysis included IA ancestry, age at blood draw, height, BMI, neighborhood SES, alcohol intake, and nativity (foreign-born vs. U.S.-born). Analyses were also adjusted for endogenous estrogen measurements.

Data on reproductive variables were available from the interview (i.e., age at menarche, age at first full term pregnancy, number of full term pregnancies). We ran multivariable models adjusting for reproductive variables, but as we had previously hypothesized, they did not have an effect on E activity at blood draw, which for most women occurred many years after the individual’s last pregnancy. Therefore, final models do not adjust for reproductive variables. Additionally, because the average age at blood draw for all three racial/ethnic groups was over the age of 60 years, which is about 10 years after the average age of menopause in U.S. women, final models did not adjust for age at menopause.

## Results

Descriptive characteristics by racial/ethnic groups are presented in Table 1. After adjusting for plate, ER RLUs were highest for NLB women (mean of 8844 RLUs), followed by NLWs (mean of 8155 RLUs), and lowest for Latina women (mean RLUs of 6226). NLW women were older at blood draw (66.8 yrs, sd. 10.5 yrs) when compared to NLB (61.7 yrs, sd. 9.8 yrs) and Latina (61.5 yrs, sd. 9.5 yrs) women. Age at menarche was similar for the three groups. NLB women had the lowest age at first full term pregnancy (21 yrs, sd. 5 yrs). However, Latina women had a greater number of full term pregnancies (3.8) compared to the NLW (2.4) and NLB women (2.8). Latina women were on average shorter than NLBs and NLWs. A large percentage (66%) of the Latina women in this study were foreign-born, while over 90% of the NLW and NLB women were U.S.-born. About 87% and 84% of the Latina and NLB women, respectively, were categorized as overweight or obese. A greater proportion of NLW women reported consuming some alcohol (54%), while 76% and 71% of NLB and Latina women, respectively, reported no alcohol intake during the year before the interview. Lastly, women in this study were predominantly postmenopausal either naturally or due to surgery. Although a statistically significant greater proportion (27%) of NLB women had a history of hysterectomy and/or oophorectomy compared to NLW and Latina women, menopausal status did not differ between the three racial/ethnic groups.

**Table 1:** Participant characteristics by racial/ethnic group (N = 503).

**Continuous variables**		Latinas			NLWs			NLBs		P value
	N	Mean	Sd.	N	Mean	Sd.	N	Mean	Sd.	
Estrogenic Activity in RLUs Median (IQR)	329	6226 2452 (1582–4478)	12899	74	8155 2248 (1722–4691)	13307	100	8844 3335 (1883–10124)	11260	0.0157
Endogenous estrogen level^a^, pg/mL Median (IQR)	327	58.9 46.7 (34.5–64.5)	49.4	74	52.1 40.9 (34.5–60.4)	32.1	100	64.0 53.6 (36.5–81.3)	38.2	0.0613
Age at blood draw (years)	329	61.5	9.5	74	66.8	10.5	100	61.7	9.8	0.0001
Age at menarche, yrs	326	12.9	1.9	74	12.8	1.5	99	12.7	1.7	0.6396
Age at first full term pregnancy, yrs	306	23.0	5.2	58	24.2	4.8	88	20.8	4.9	0.0001
Number of full term pregnancies	329	3.8	2.7	74	2.4	1.8	100	2.8	2.2	<0.0001
Height (cm)	324	155.5	6.9	73	161.1	7.3	100	163.3	6.2	<0.0001
Individual African Ancestry proportion (%)	285	8	7							
Individual Indigenous American ancestry proportion (%)	285	41	15							
**Categorical variables**	Latinas			NLWs			NLBs			P value
	N	%		N	%		N	%		
Nativity										
U.S.-born	112	34		68	92		97	97		<0.0001
Foreign-born	217	66		6	8		3	3		
Family history of breast cancer^b^										
No	290	88		62	84		88	88		0.559
Yes	39	12		12	16		12	12		
Alcohol intake (gms per day)^c^										
None	235	71		34	46		76	76		<0.0001
<10	82	25		20	27		15	15		
≥10	12	4		20	27		9	9		
BMI (kg/m^2^)										
<25	42	13		22	30		16	16		<0.0001
25–29.9	122	38		30	41		24	24		
≥30	160	49		21	29		60	60		
Age at blood draw (years)										
<55	94	29		14	19		31	31		0.001
55–65	117	36		13	18		31	31		
≥66	118	36		47	64		38	38		
Menopausal status at blood draw										
Premenopausal	28	9		8	11		6	7		0.571
Postmenopausal	274	91		63	89		86	93		
History of oophorectomy and/or hysterectomy	46	14		10	14		27	27		0.010
Neighborhood socioeconomic status (SES)										
1 (low SES)	17	5		3	4		23	23		<0.0001
2	77	24		6	8		30	30		
3	91	28		12	16		25	25		
4	80	25		21	29		11	11		
5 (high SES)	61	19		31	42		10	10		

NLW, Non Latina White; NLB, Non Latina Black; RLUs, relative light units reported by bioassays (untransformed)

^a^ Sum of endogenous estrogens including estrone and bioavailable estradiol (free and albumin bound)

^b^ First degree relatives

^c^ Alcohol intake during calendar year before selection into parent study

In the univariate model (Table 2), Latinas had 13% lower E activity (p = 0.239) and NLBs had 35% higher activity (p = 0.04) compared to NLWs. After adjusting for endogenous estrogens, the trend was consistent with 11% higher E activity in NLB women and 21% lower activity in Latina women; however, the association was statistically significant only for Latina women (p = 0.019). In the multivariable model, Latinas showed lower E activity before (26%, p = 0.026) and after (25%, p = 0.01) adjusting for endogenous estrogens. Although E activity in NLBs continued to remain higher than that of NLWs, the difference between the two groups was no longer statistically significant before (13%, p = 0.431) or after (5%, p = 0.673) adjustment for endogenous estrogens. We observed a decrease in E activity with increasing age, which was no longer statistically significant after adjustment for endogenous estrogens. In unadjusted models, E activity was 67% higher for obese women (p<0.001) and 40% higher for overweight women (p = 0.006) when compared to those with normal BMI (p<0.001), but these associations were no longer apparent after adjusting for endogenous estrogen levels. Overall, no statistically significant associations were observed with neighborhood SES before or after adjustment for endogenous estrogens. Compared to women who reported no alcohol consumption, those who consumed <10gms of alcohol had lower E activity before (21%, p = 0.017) and after (17%, p = 0.024) adjusting for endogenous estrogens.

**Table 2:** Association of estrogenic activity with lifestyle and demographic factors in all women (N = 488) using univariate and multivariable analyses.

Univariate	Coefficient^a^ (95% CI)	%Change in RLUs*	P value	Adjusted by endogenous estrogens^b^	%Change in RLUs*	P value
Race/ethnicity^#^						
NLW	ref			ref		
NLB	0.30 (0.01, 0.58)	35	0.04	0.10 (-0.13, 0.32)	11	0.398
Latina	-0.14 (-0.38, 0.10)	-13	0.239	-0.23 (-0.42, -0.04)	-21	0.019
**Multivariable**						
Race/ethnicity						
NLW	ref			ref		
NLB	0.12 (-0.18, 0.43)	13	0.431	0.05 (-0.20, 0.31)	5	0.673
Latina	-0.30 (-0.57, -0.04)	-26	0.026	-0.29 (-0.50, -0.07)	-25	0.01
Age at blood draw (years)						
<55	ref			ref		
55–65	-0.32 (-0.53, -0.11)	-27	0.003	-0.01 (-0.19, 0.16)	-1	0.884
>65	-0.41 (-0.62, -0.21)	-34	<0.001	-0.06 (-0.24, 0.11)	-6	0.491
Height, cm	0.01 (-0.01, 0.02)	1	0.33	0.00 (-0.01, 0.01)	0	0.684
BMI (kg/m^2^)						
<25	ref			ref		
25–29.9	0.34 (0.10, 0.59)	40	0.006	0.12 (-0.10, 0.32)	13	0.272
≥30	0.51 (0.27, 0.75)	67	<0.001	0.13 (0.08, 0.33)	14	0.225
Neighborhood SES						
1 (low)	ref			ref		
2	0.27 (-0.06, 0.60)	31	0.114	0.12 (-0.15, 0.39)	13	0.39
3	0.10 (-0.23, 0.43)	11	0.565	0.01 (-0.26, 0.28)	1	0.915
4	0.31 (-0.03, 0.65)	36	0.078	0.20 (-0.08, 0.48)	22	0.163
5 (high)	0.25 (-0.10, 0.60)	28	0.157	0.10 (-0.18, 0.39)	11	0.475
Alcohol intake (gms/day)^c^						
None	ref			ref		
<10	-0.24 (-0.44, -0.04)	-21	0.017	-0.19 (-0.35, -0.02)	-17	0.024
≥10	-0.25 (-0.57, 0.07)	-22	0.122	-0.20 (-0.46, 0.06)	-18	0.129

CI, confidence interval

^#^ ANOVA p-value = 0.002

* Luciferase reporter assay results expressed in relative light units (RLUs). Percent change in RLUs (untransformed) per unit change in predictor was estimated using the formula [e ^β^ − 1] * 100.

^a^ The coefficients and 95% CI are based on the ln-transformed RLUs

^b^ Sum of endogenous estrogens including estrone and bioavailable estradiol (free and albumin bound)

^c^ Alcohol intake during calendar year before selection into parent study

Restricting the analysis to Latina women with information on genetic ancestry (N = 276), we found that in the univariate model higher IA ancestry was associated with lower E activity (Table 3). This finding was statistically significant prior to adjusting for endogenous estrogens (52%, p = 0.031) and marginally significant after adjustment (39%, p = 0.088). In the multivariable analysis, increasing age was associated with lower E activity. As with the full sample, the significant associations with BMI were no longer statistically significant after adjusting for endogenous estrogens. No statically significant associations were observed for height, neighborhood SES or nativity.

**Table 3:** Association between estrogenic activity and Indigenous American ancestry in Latina women (N = 276) using univariate and multivariable analysesUnivariate.

Univariate	Coefficient (95% CI) ^a^	%Change in RLUs*	P value	Coefficient (95% CI) ^a^	%Change in RLUs*	P value
Indigenous American ancestry	-0.74 (-1.41, -0.07)	-52	0.031	-0.49 (-0.06, 0.07)	-39	0.088
**Multivariable**		**Model A**^**b**^		**Model A + endogenous estrogens**		
Indigenous American ancestry	-1.10 (-1.84, -0.35)	-67	0.004	-0.71 (-1.34, -0.081)	-51	0.027
Age at blood draw (years)						
<55	ref			ref		
55–65	-0.28 (-0.55, -0.01)	-24	0.041	-0.01 (-0.24, 0.22)	-1	0.918
>65	-0.37 (-0.64, -0.10)	-31	0.008	-0.05 (-0.28, 0.19)	-5	0.681
Height, cm	-0.00 (-0.02, 0.01)	0	0.58	-0.01 (-0.02, 0.01)	-1	0.431
BMI (kg/m^2^)						
<25	ref			ref		
25–29.9	0.49 (0.15, 0.84)	63	0.005	0.25 (-0.05, 0.54)	28	0.107
≥30	0.47 (0.13, 0.82)	60	0.006	0.17 (-0.12, 0.47)	19	0.251
Neighborhood SES						
1 (low)	ref			ref		
2	0.17 (-0.32, 0.67)	19	0.491	0.01 (-0.41, 0.43)	1	0.956
3	-0.02 (-0.50, 0.47)	-2	0.948	-0.03 (-0.44, 0.38)	-3	0.887
4	0.23 (-0.27, 0.73)	26	0.364	0.12 (-0.30,0.54)	13	0.589
5 (high)	0.18 (-0.33, 0.70)	20	0.49	0.09 (-0.35, 0.52)	9	0.697
Alcohol intake (gms/day)^c^						
None	ref					
<10	-0.35 (-0.59, -0.10)	-30	0.007	-0.24 (-0.45, -0.03)	-21	0.026
≥10	-0.16 (-0.69, 0.38)	-15	0.561	-0.15 (-0.60, 0.30)	-14	0.511
Foreign born						
Yes	-0.02 (-0.26, 0.22)	-2	0.899	-0.03 (-0.23, 0.17)	-3	0.779

CI, confidence interval

* Luciferase reporter assay results expressed in relative light units (RLUs). Percent change in RLUs (untransformed) per unit change in predictor was estimated using the formula [e ^β^ − 1] * 100.

^a^ The coefficients and 95% CI are based on the ln-transformed RLUs

^b^ Unadjusted for endogenous estrogen levels

^c^ Alcohol intake during calendar year before selection into parent study

## Discussion

Our study shows that there are significant differences in E activity in women across three racial/ethnic groups, partly due to differences in BMI (in the case of NLWs vs. NLBs) and potential exposure to exogenous estrogen-like compounds (NLWs vs. Latinas). Results also suggest that higher IA ancestry in Latina women is associated with lower levels of E activity, which is consistent with the observation that Latina women with high IA ancestry have lower risk of developing breast cancer.

There was an association between BMI and E activity in all racial/ethnic groups. In NLB women, higher E activity was mostly attributed to BMI. After inclusion of the BMI variable in the multivariable model, the level of E activity among NLBs was no longer statistically different from NLWs. The attenuation of the positive association between E activity and BMI after adjusting for endogenous estrogen levels in the regression model is consistent with previous observations [23–25]. In overweight and obese postmenopausal women, the adipose tissue serves as the primary source of estrogen synthesis and leads to elevated estrogen measures. This is likely to be one of multiple mechanisms underlying the association between BMI and breast cancer risk in postmenopausal women [11]. Interestingly, weight loss interventions [26] in postmenopausal women have been associated with lower circulating estradiol, suggesting a reversal of peripheral production of estrogens via reduction of adipose tissue. Since the association between E activity and BMI is present in all racial/ethnic groups, a reduction of E activity through weight management could lead to breast cancer risk reduction in all women. The lower E activity among Latinas, especially among Latina women with high IA ancestry, was not fully explained by the relationship between BMI and endogenous estrogen levels and should be further investigated.

Another lifestyle factor that showed association with E activity was alcohol intake. Alcohol intake is associated with risk of several cancers including breast cancer. However, studies looking at the effects of alcohol consumption on circulating levels of endogenous estrogen have produced inconsistent results. Some studies reported that increased alcohol consumption is associated with higher circulating estrogens [27], while others found no association [28]. Our results are not consistent with previous findings, given that our data show an inverse association between E activity and alcohol consumption. More studies that include samples from diverse populations are needed to identify the effects of alcohol consumption on endogenous estrogen levels.

The association between IA ancestry and E activity among Latinas even after adjusting for endogenous estrogens suggests that in this group, the E activity could be related to environmental exposures such as exogenous chemicals or dietary constituents. Soy-based and vegetable-derived foods containing several phytoestrogens are common in the diet of Latino populations [29]. Because phytoestrogens have a similar chemical structure to estradiol, they may compete with estrogens for binding to ER. Dietary lignan consumption has been found to be associated with reduced risk of postmenopausal breast cancer specifically in ER and progesterone receptor-positive cases, suggesting that these compounds are acting through an ER related mechanism [30]. However, this hypothesis needs to be further investigated. Another explanation for the lower E activity among Latina women with high IA ancestry after adjusting for endogenous estrogen levels would be a biological difference in the metabolism of estrogen and estrogen-like compounds, resulting in a fewer number of molecules that could adequately bind to the ER [31]. Some studies have demonstrated that estrogen metabolism and hydroxylation of parent estrogen compounds at different positions around the steroid ring, mainly 2-hydroxylation, is associated with reduced risk of postmenopausal breast cancer [32].

An important strength of the present study is the diverse study population and the larger sample set of Latina women included in the analysis of IA ancestry (n = 276) compared to our previous pilot study that included only 90 Mexican women [18]. Using this larger sample size, we have validated our previous findings of a negative association between E activity and IA ancestry. The association between endogenous estrogen levels and IA ancestry was statistically significant and could partly explain why Latina women with higher IA ancestry have a lower incidence of breast cancer. Furthermore, we were able to assess E activity using a commonly used bioassay and correlate measures with endogenous estrogen levels. The Pearson correlation coefficient between the two methods was high (0.62, p<0.0001). Although the methods were not perfectly correlated, our results highlight the potential role of exogenous estrogenic compounds in the activation of the ER and the concomitant effects on the endocrine system. More importantly, given that most breast cancer grows in the presence of estrogens, understanding what factors (of endogenous and exogenous origin) might stimulate or block the estrogen receptor among women of different racial/ethnic backgrounds is of great relevance.

Our study also has some limitations. Although we were able to assess total E activity in plasma samples and account for endogenous estrogens, this approach does not allow us to determine the specific estrogen-like compounds that are acting on the ER. However, we report total E activity, which encompasses both endogenous and exogenous estrogens and is a comprehensive representation of estrogenic exposure. Additionally, our results are based on blood samples from a single time point, which may not represent the fluctuations of estrogens over time. Yet, the endogenous estrogen levels in postmenopausal women are relatively stable over time [33] and fluctuations in total estrogens will be mostly due to exogenous estrogens that are captured by the bioassay. Our study only included control women; assessing associations with breast cancer risk using a case-control design is not feasible because measuring E activity in plasma from breast cancer cases would not reflect E activity before diagnosis. However, the association between levels of circulating estrogens and breast cancer risk is well established [31], highlighting the significance of the associations reported in the present study for breast cancer risk. Further replication of the association between race/ethnicity, BMI, genetic ancestry, E activity, endogenous hormones and breast cancer risk in a prospective cohort with information about possible sources of exogenous estrogens (i.e., diet) and genetic data will provide the ideal setting to investigate the role that estrogen-related factors play in explaining differences in breast cancer incidence between racial/ethnic groups. Such knowledge could lead to race/ethnicity/ancestry-specific interventions focused on lowering E activity to reduce the risk of developing breast cancer in all racial/ethnic groups.

## Conclusions

Given the central role of the activation of the ER in the etiology of breast cancer, a better understanding of the receptor’s interaction with the receptor ligands, whether endogenous or exogenous, is imperative. Additionally, the discovery of specific compounds that are modulating the receptor and are present at different levels in different populations, would lead to changes in exposure to these compounds and ultimately, to changes in breast cancer risk.

## Supporting information

S1 Fig Unadjusted estrogenic activity in all women (N = 503). (PDF)

S1 Dataset Covariate information used in this study. (XLS)
