# Supplementary figures and images for: Estrogenic activity, race/ethnicity, and Indigenous American ancestry among San Francisco Bay Area women
Source: PLoS One. 2019 Mar 25;14(3):e0213809. doi: 10.1371/journal.pone.0213809 (PMC6433244; doi:10.1371/journal.pone.0213809)

**S1 Figure. Unadjusted estrogenic activity in all women (N=503)**

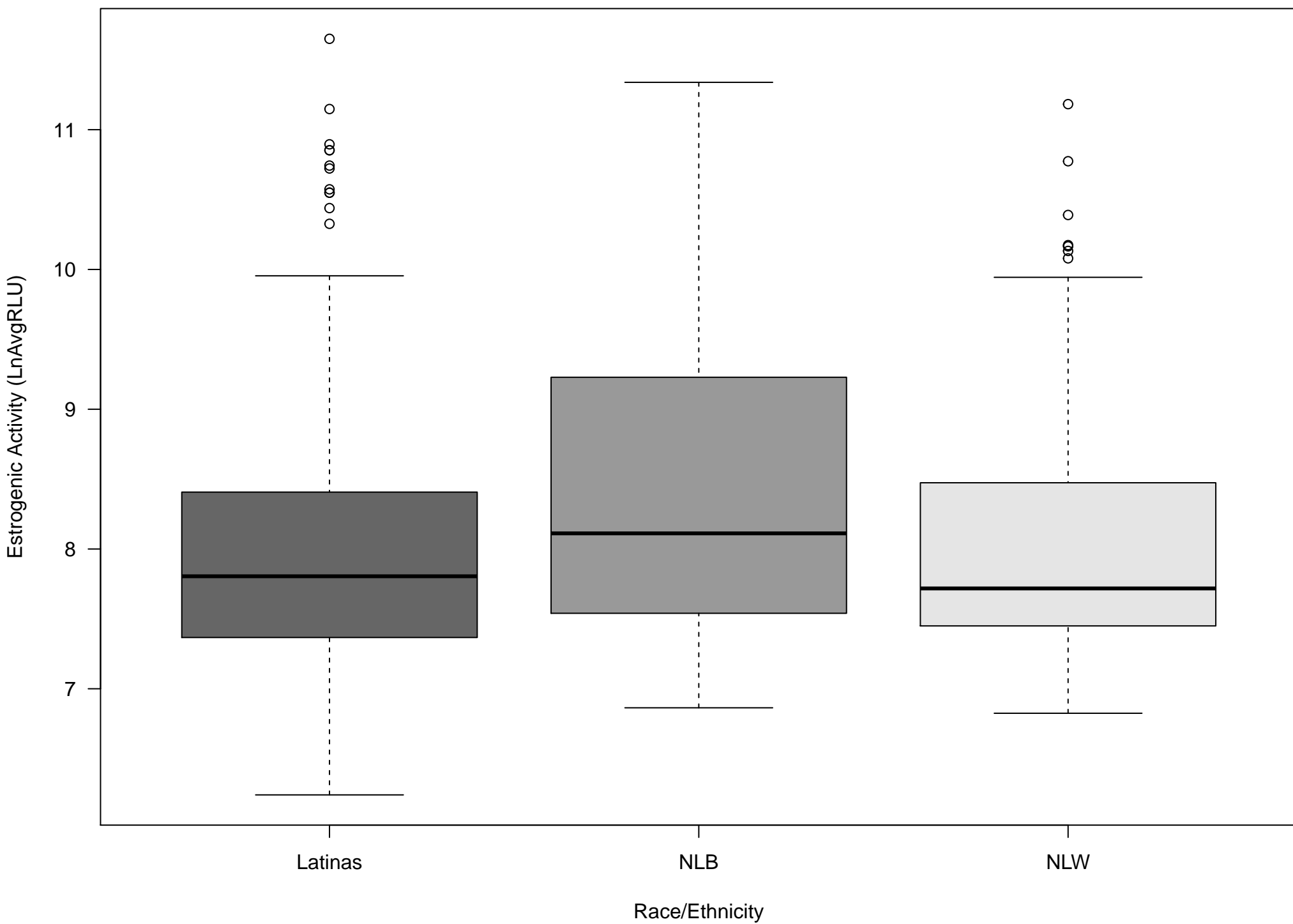

Supplement: S1 Fig — (PDF) [file pone.0213809.s001.pdf]
